# Determination of methotrexate using carbon paste electrode modified with ionic liquid/Ni-Co layered double hydroxide nanosheets as a voltammetric sensor

**DOI:** 10.5599/admet.2460

**Published:** 2024-09-04

**Authors:** Peyman Mohammadzadeh Jahani, Fariba Garkani Nejad, Reza Zaimbashi, Mohammad Reza Aflatoonian, Somayeh Tajik, Hadi Beitollahi

**Affiliations:** 1 School of Medicine, Bam University of Medical Sciences, Bam, Iran; 2 Environment Department, Institute of Science and High Technology and Environmental Sciences, Graduate University of Advanced Technology, Kerman, Iran; 3 Leishmaniasis Research Center, Kerman University of Medical Sciences, Kerman, Iran; 4 Research Center of Tropical and Infectious Diseases, Kerman University of Medical Sciences, Kerman, Iran

**Keywords:** Electrochemical sensor, real sample analysis, cyclic voltammetry, differential pulse voltammetry

## Abstract

**Background and purpose:**

Methotrexate (MTX) is a widely used anti-cancer drug, but its overuse can lead to significant side effects. Therefore, it is very vital to design simple and sensitive analytical methods for its determination.

**Experimental approach:**

In this work, an electrochemical sensor was prepared based on an ionic liquid (IL)/Ni-Co layered double hydroxide nanosheets (Ni-Co-LDH)-modified carbon paste electrode IL/Ni-Co-LDH/CPE. Cyclic voltammetry, differential pulse voltammetry, and chronoamperometry methods were applied to evaluate the performance of the designed sensor for MTX determination.

**Key results:**

The IL/Ni-Co-LDH/CPE sensor exhibits a linear relationship between the peak current of the differential pulse voltammetry and MTX concentrations in the linear dynamic range of 0.02 to 140.0 μM, with a detection limit of 0.006 μM. The IL/Ni-Co-LDH/CPE sensor exhibited relative standard deviation values between 1.7 to 3.7 % for recovery tests on real samples, indicating the precision of the method.

**Conclusion:**

The designed sensor with cost-effective and good performance could be valuable for therapeutic drug monitoring and clinical diagnostics.

## Introduction

Methotrexate (MTX) is a widely used anti-cancer drug. Its mechanism of action involves inhibiting the formation of tetrahydrofolate. As a result, it blocks the growth of tumour cells. MTX has been used effectively to treat a variety of cancers, such as head and neck cancer, graft-versus-host disease, and childhood acute leukaemia. However, the clinical use of MTX is limited due to the risk of severe side effects, including hepatotoxicity, low white blood cell counts, and ulcerative stomatitis. Given the therapeutic importance of MTX and the need to manage its side effects, the development of a sensitive, accurate, and rapid detection method is crucial for clinical applications and pharmacological research. Accurate monitoring of MTX levels can help clinicians optimize dosages, minimize adverse effects, and improve patient outcomes during MTX treatment [1-3]. Over time, researchers have utilized various analytical techniques to detect and quantify MTX concentrations. Some commonly used methods include spectrophotometry, high-performance liquid chromatography, capillary electrophoresis, chemiluminescence, and liquid chromatography tandem mass spectrometry [4-8].

Electrochemical techniques offer several advantages for the detection and quantification of target species [9-20]. These advantages include high sensitivity, good selectivity, rapid methodologies, portability, and economical and simple operation [21-26]. However, the direct electrooxidation of target species at bare electrode surfaces may not be suitable for analytical applications due to high overpotentials and slow electrode kinetics. To address this, researchers have developed and utilized various chemically modified electrodes (CMEs) [27-32]. The use of CMEs can significantly lower the overpotentials and increase the oxidation current response, thereby improving the analytical performance for the detection of target analytes [33-38].

The choice of the appropriate electrode modification material, which should possess good conductivity and electrochemical properties, is crucial for the development of highly precise and sensitive analytical methods for the effective detection of MTX and other target analytes. Nanotechnology has been recognized as one of the most important technologies in recent years, which has received much attention due to its wide applications in various fields [39-49]. Especially, nanostructures have attractive properties for electrochemical sensing applications [50-55].

Layered double hydroxides (LDHs) have emerged as promising two-dimensional host/guest nanomaterials with a wide range of applications, including adsorption, catalysis, supercapacitors, fire retardants, electrochemical sensors, and biochemical applications. LDHs are composed of divalent and trivalent metal ions, where the divalent metal ions (*e.g*., Mg^2+^) can be substituted by other divalent ions, such as Al, Ni, Mn, Co, or Cu, with a similar ionic radius. This versatility in metal composition allows for tailoring LDH properties to suit specific applications. The open and layered structure of LDHs, combined with their high biocompatibility, make them attractive materials for use as stable matrices or effective redox mediators in diverse electrochemical sensor applications. In these contexts, LDHs exhibit exceptional catalytic properties that can enhance the sensitivity and performance of electrochemical sensing platforms. The unique structural and chemical characteristics of LDHs, such as their tunable composition, high surface area, and ion-exchange capabilities, have contributed to their growing popularity in the development of advanced electrochemical sensors. Researchers have actively explored the integration of LDHs into sensor designs to leverage their unique properties and improve the overall analytical performance for the detection of various target analytes. The versatility and advantageous properties of LDHs have led to their increasing adoption in a wide range of electrochemical sensing applications, making them a valuable class of materials for the advancement of sensitive, selective, and robust analytical techniques [56-59].

Ionic liquids (ILs) are characterized by low melting points, often at or below ambient temperature. In recent years, ILs have gained significant attention as a frontier and novel area of research due to their unique chemical and physical properties. Some of the key characteristics of ILs include negligible vapor pressure, high conductivity, wide electrochemical windows, and high chemical and thermal stability. Recently, ILs have been proposed as efficient binders for the preparation of ionic liquid-based paste electrodes. The unique properties of ILs, make them attractive candidates for the development of advanced electrochemical sensing platforms. The use of ionic liquid-based paste electrodes has been explored in various analytical applications, taking advantage of the enhanced stability, conductivity, and electrochemical performance provided by the incorporation of ILs. This has led to the emergence of ILs as an innovative and promising class of materials in electrochemical sensors and electroanalytical techniques [60-63].

Based on the previous information provided, the current study focused on developing an advanced electrochemical sensor for the determination of MTX. In the first part of the study, the electrochemical behaviour and detection of MTX was investigated using a bare carbon paste electrode (CPE). However, the direct electrooxidation of MTX at the bare CPE surface exhibited some limitations, such as slow electrode kinetics and high overpotentials. To address these limitations, we focused on the preparation of the innovative electrochemical sensor by combining the advantageous properties of ILs and layered double hydroxides (LDHs). Specifically, an IL/Ni-Co-LDH-based sensor (IL/Ni-Co-LDH/CPE) was fabricated and its performance in the determination of MTX was evaluated. It is expected that the integration of IL and Ni-Co-LDH in the sensor design contributed to the enhanced electrochemical performance, leveraging the unique properties of these materials, such as high conductivity, catalytic activity, and efficient electron transfer kinetics.

## Experimental

### Reagents and chemicals

Graphite and liquid paraffin were purchased from Sigma Aldrich and Merck, respectively. Methotrexate (MTX) and other chemicals were of analytical grade and used without further purification. PBS was used as supporting electrolyte. All the electrochemical measurements and data collection were performed at room temperature (25 °C).

**Figure 1.**
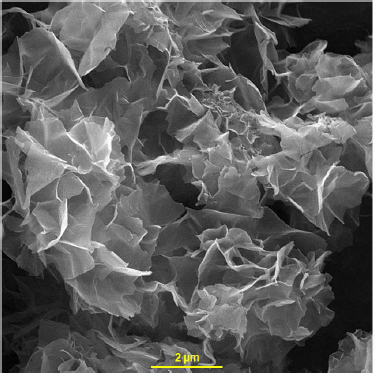
FE-SEM image of Ni-Co LDH nanosheets.

The synthesis and characterization of Ni-Co LDH nanosheets were described in our previous work [64]. Figure 1 shows the FE-SEM image of Ni-Co LDH nanosheets.

### Preparation of modified electrode

The bare CPE was prepared by thoroughly mixing graphite powder and liquid paraffin in a mortar and pestle. The composite mixture was then packed into the end of a glassy tube. Electrical contact was made by inserting a copper wire into the back of the composite mixture in the glassy tube.

The modified electrode, IL/Ni-Co-LDH/CPE, was prepared by mixing the unmodified CPE composite (graphite and liquid paraffin) with ionic liquid (IL) (1-butyl-3-methylimidazolium hexafluorophosphate) and nickel-cobalt layered double hydroxide (Ni-Co-LDH) using the same method as the bare CPE. The resulting IL/Ni-Co-LDH/CPE was then packed into the end of a glassy tube, and electrical contact was established by inserting a copper wire into the back of the composite.

## Results and discussion

### Electrochemical behavior of MTX

**Figure 2.**
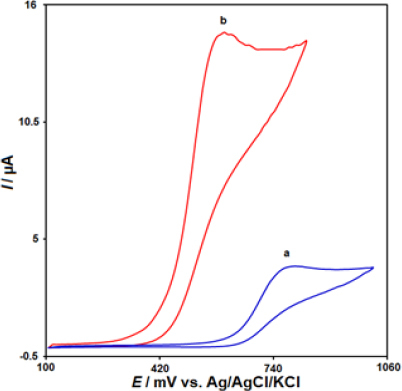
The CVs of 40.0 μM MTX solution (pH 7.0) at the surface of a bare CPE (a) and the modified IL/Ni-Co-LDH/CPE electrode (b).

The electrochemical behaviour of MTX was investigated using cyclic voltammetry (CV) at different electrode surfaces in a 0.1 M PBS at pH 7.0, as shown in Figure 2. At the unmodified CPE, a small oxidation peak was observed with a peak current of 3.7 μA and a peak potential of 790 mV (curve a). In contrast, at the IL/Ni-Co-LDH modified electrode, the peak potential was negatively shifted to 600 mV, and the peak current was approximately 4 times higher than that of the bare CPE (curve b). This significant enhancement in the peak current was attributed to the combined effects of the electron acceleration and catalytic properties of the IL/Ni-Co-LDH/CPE modifiers, which acted synergistically to improve the electrochemical detection of MTX. The results clearly demonstrate that the IL/Ni-Co-LDH/CPE sensor exhibited excellent electrocatalytic performance towards the oxidation of MTX compared to the unmodified CPE. Therefore, all the remaining electrochemical studies were conducted using the IL/Ni-Co-LDH/CPE sensor to take advantage of its superior sensitivity for the determination of MTX.

### Influence of scan rate

**Figure 3.**
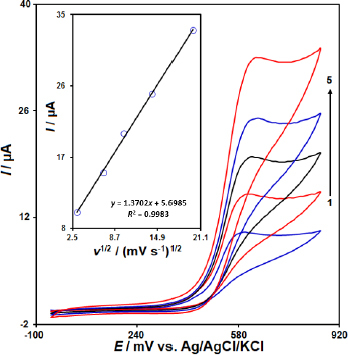
The CVs of 40.0 μM MTX solution in PBS (0.1 M, pH 7.0) at the surface of the IL/Ni-Co-LDH/CPE at different scan rates: (1) 10, (2) 50, (3) 100, (4) 200 and (5) 400 mV/s.

Figure 3 illustrates the impact of varying scan rates on the oxidation currents of MTX. Increasing the scan rate led to an enhancement of the peak currents. Moreover, the linear relationship between the *I*_p_ and the square root of the scan rate (*ν*^1/2^) suggests that the oxidation process is diffusion-controlled (Figure 3 inset). This finding is significant, as it provides insights into the mechanism of the electrochemical detection of MTX at the IL/Ni-Co-LDH/CPE sensor and supports the excellent electrocatalytic properties of the IL/Ni-Co-LDH/CPE towards the oxidation of MTX.

### Chronoamperometry

**Figure 4.**
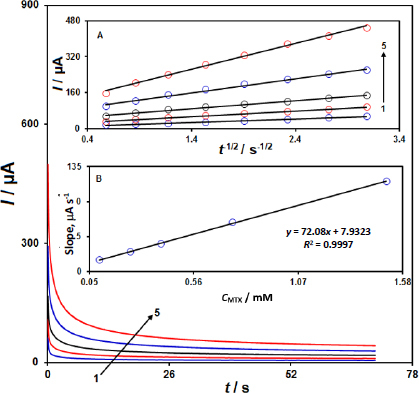
Chronoamperograms at the IL/Ni-Co-LDH/CPE in the presence of different concentrations of MTX: 0.1(1), 0.25(2), 0.4(3), 0.75(4), and 1.5(5) mM in PBS (0.1 M, pH 7.0). Inset A: the plots of current (*I*) versus the square root of time (*t*^-1/2^) from the chronoamperometry data. Inset B: Slope plot of the straight lines versus MTX concentrations.

The diffusion coefficient (*D*) of MTX was determined using chronoamperometry experiments at the IL/Ni-Co-LDH/CPE sensor. Figure 4 shows the chronoamperograms recorded for different concentrations of MTX (0.1 to 1.5 mM) at a step potential of 650 mV. The slopes of the Cottrell plots (current vs. the inverse square root of time) for MTX, as shown in Figure 4A, were used to calculate the diffusion coefficient according to the Cottrell equation. Using the slope obtained from the plots and the Cottrell equation, the diffusion coefficient (*D*) of MTX was calculated to be 5.4×10^-5^ cm^2^/s.

This value of the diffusion coefficient provides important information about the mass transport characteristics of MTX at the IL/Ni-Co-LDH/CPE sensor, which is relevant for understanding the electrochemical behaviour and the sensor performance.

### Electrochemical determination of MTX at IL/Ni-Co-LDH/CPE

**Figure 5.**
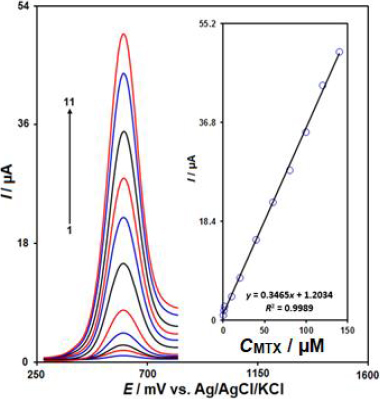
DPVs of the IL/Ni-Co-LDH/CPE in solutions containing different concentrations of MTX: (1) 0.02, (2) 0.8, (3) 2.0, (4) 10.0, (5) 20.0, (6) 40.0, (7) 60.0, (8) 80.0, (9) 100.0, (10) 120.0 and (11) 140.0 μM in PBS (0.1 M, pH 7.0). Inset: the plot of the *I* as a function of MTX concentration.

Figure 5 displays the differential pulse voltammetry (DPV) curves corresponding to the electrocatalytic oxidation of MTX at the IL/Ni-Co-LDH/CPE, recorded at a scan rate of 50 mV/s. As shown in Figure 5, the oxidation peak of MTX was observed at a potential of 600 mV. Upon increasing the concentrations of MTX in 0.1 M PBS (pH 7.0), the oxidation peak currents increased linearly. The inset in Figure 5 shows the calibration plot of the oxidation peak current versus the MTX concentrations. The oxidation current had a linear relationship with MTX concentrations ranging from 0.02 to 140.0 μM, (*R*^2^ = 0.9989). The LOD (3*S*_b_/*m*) for MTX was calculated to be 0.006 μM. The sensitivity of the IL/Ni-Co-LDH/CPE sensor towards the electrochemical detection of MTX was found to be 0.3465 μA/μM. These results demonstrate the excellent analytical performance of the IL/Ni-Co-LDH/CPE sensor for the sensitive determination of MTX, which can be attributed to the synergistic effects of the ionic liquid and the Ni-Co-LDH modifiers on the electrochemical oxidation of MTX.

### Determination of MTX in MTX tablets and urine samples

To assess the applicability of the proposed IL/Ni-Co-LDH/CPE sensor, it was used for the determination of MTX content in commercial pharmaceutical preparations and urine samples using standard addition method. The obtained results are given in Table 1. The recoveries demonstrate a very good accuracy of the proposed electrochemical method for the determination of MTX in both clinical preparations and biological fluids. These results indicate that the IL/Ni-Co-LDH/CPE sensor can be successfully applied for the sensitive and accurate determination of MTX in pharmaceutical formulations and clinical samples, with good reliability.

## Conclusion

In this study, a novel electrochemical sensor for the detection of MTX was successfully developed using IL and Ni-Co-LDH as the modifiers. The Ni-Co-LDH material provided good properties, which enhanced the electrochemical performance of the sensor. The proposed IL/Ni-Co-LDH/CPE sensor exhibited a wide LDR of 0.02 to 140.0 μM and a remarkably low LOD of 0.006 μM for MTX. This study presents an innovative strategy for the sensitive electrochemical detection of MTX, which leverages the synergistic effects of IL and Ni-Co-LDH as the sensing platform. The remarkable analytical performance demonstrates the potential of combining these two functional materials in an electrochemical assay for the rapid and reliable quantification of MTX. The developed IL/Ni-Co-LDH/CPE sensor showed great promise for practical applications in the determination of MTX in clinical preparations and pharmaceutical formulations, with high sensitivity, accuracy, and reliability.

## Figures and Tables

**Table 1.** The application of ILs/Ni-Co-LDH/CPE for determination of MTX in real samples (*n*=5).

Sample	MTX concentration, MM		Recovery, %	RSD, %
	Spiked	Found		
MTX tablet	0.0	8.0	-	1.9
	1.0	8.9	98.9	3.2
	2.0	10.1	101.0	2.7
	3.0	11.3	102.7	2.1
	4.0	11.9	99.2	3.6
Urine	0.0	-	-	-
	4.5	4.7	104.4	3.7
	6.5	6.3	96.9	2.2
	8.5	8.6	101.2	1.7
	10.5	10.4	99.0	2.8

## References

[1] Tarlekar P. Chatterjee S. Dendritic platinum nanoparticles decorated electrochemical sensor for immensely sensitive determination of antineoplastic drug methotrexate. Diamond and Related Materials 148 (2024) 111417. https://doi.org/10.1016/j.diamond.2024.111417 10.1016/j.diamond.2024.111417

[2] Akhter S. Shalauddin M. Ahmed S.R. Lee V.S. Ibrahim F. Srinivasan S. Rajabzadeh A.R. Basirun W.J. Bio-synthesized copper nanoparticle anchored ultrathin petal-shaped black phosphorous nanosheets and 3D graphene decorated nanocomposite for electrochemical sensing of methotrexate and paracetamol in diverse matrices. Electrochimica Acta 497 (2024) 144554. https://doi.org/10.1016/j.electacta.2024.144554 10.1016/j.electacta.2024.144554

[3] Adeniyi K.O. Osmanaj B. Manavalan G. Mikkola J.P. Berisha A. Tesfalidet S. Reagentless impedimetric immunosensor for monitoring of methotrexate in human blood serum using multiwalled carbon nanotube@ polypyrrole/polytyramine film electrode. Talanta 268 (2024) 125316. https://doi.org/10.1016/j.talanta.2023.125316 10.1016/j.talanta.2023.125316 37864856

[4] Sastry C.S. Lingeswara Rao J.S. Spectrophotometric methods for the determination of methotrexate in pharmaceutical formulations. Analytical Letters 29(29) (1996) 1763-1778. https://doi.org/10.1080/00032719608001522 10.1080/00032719608001522

[5] Liu X. Liu J. Huang Y. Zhao R. Liu G. Chen Y. Determination of methotrexate in human serum by high-performance liquid chromatography combined with pseudo template molecularly imprinted polymer. Journal of Chromatography A 1216(1216) (2009) 7533-7538. https://doi.org/10.1016/j.chroma.2009.06.018 10.1016/j.chroma.2009.06.018 19559443

[6] Szakács Z. Noszál B. Determination of dissociation constants of folic acid, methotrexate, and other photolabile pteridines by pressure-assisted capillary electrophoresis. Electrophoresis 27(27) (2006) 3399-3409. https://doi.org/10.1002/elps.200600128 10.1002/elps.200600128 16944455

[7] Song Z. Wang Y. Dong Y. Xu K. Long H. Deng C. Yin Y. Eremin S.A. Meng M. Xi R. A validated chemiluminescence immunoassay for methotrexate (MTX) and its application in a pharmacokinetic study. Analytical Methods 8(8) (2016) 162-170. https://doi.org/10.1039/C5AY02270C 10.1039/C5AY02270C

[8] Al-Ghobashy M.A. Hassan S.A. Abdelaziz D.H. Elhosseiny N.M. Sabry N.A. Attia A.S. El-Sayed M.H. Development and validation of LC-MS/MS assay for the simultaneous determination of methotrexate, 6-mercaptopurine and its active metabolite 6-thioguanine in plasma of children with acute lymphoblastic leukemia: Correlation with genetic polymorphism. Journal of Chromatography B 1038 (2016) 88-94. https://doi.org/10.1016/j.jchromb.2016.10.035 10.1016/j.jchromb.2016.10.035 27802917

[9] Mutharani B. Ranganathan P. Chen S.M. Sireesha P. Ultrasound-induced radicals initiated the formation of inorganic-organic Pr2O3/polystyrene hybrid composite for electro-oxidative determination of chemotherapeutic drug methotrexate. Ultrasonics sonochemistry 56 (2019) 410-421. https://doi.org/10.1016/j.ultsonch.2019.04.029 10.1016/j.ultsonch.2019.04.029 31101279

[10] Wei Y. Luo L. Ding Y. Si X. Ning Y. Highly sensitive determination of methotrexate at poly(l-lysine) modified electrode in the presence of sodium dodecyl benzene sulfonate. Bioelectrochemistry 98 (2014) 70-75. https://doi.org/10.1016/j.bioelechem.2014.03.005 10.1016/j.bioelechem.2014.03.005 24727063

[11] Deng Z. Li H. Tian Q. Zhou Y. Yang X. Yu Y. Jiang B. Xu Y. Zhou T. Electrochemical detection of methotrexate in serum sample based on the modified acetylene black sensor. Microchemical Journal 157 (2020) 105058. https://doi.org/10.1016/j.microc.2020.105058 10.1016/j.microc.2020.105058

[12] Yamuna A. Chen T.W. Chen S.M. Yu M.C. Yu J. One-pot synthesis of antimony oxide and bismuth oxide nanocomposites for the selective electrochemical determination of the anti-cancer drug methotrexate in biomedical samples. Ceramics International 48(48) (2022) 2369-2376. https://doi.org/10.1016/j.ceramint.2021.10.017 10.1016/j.ceramint.2021.10.017

[13] Hareesha N. Manjunatha J.G. Alothman Z.A. Sillanpää M. Simple and affordable graphene nano-platelets and carbon nanocomposite surface decorated with cetrimonium bromide as a highly responsive electrochemical sensor for rutin detection. Journal of Electroanalytical Chemistry 917 (2022) 116388. https://doi.org/10.1016/j.jelechem.2022.116388 10.1016/j.jelechem.2022.116388

[14] Tajik S. Beitollahi H. Shahsavari S. Nejad F.G. Simultaneous and selective electrochemical sensing of methotrexate and folic acid in biological fluids and pharmaceutical samples using Fe_3_O_4_/ppy/Pd nanocomposite modified screen printed graphite electrode. Chemosphere 291 (2022) 132736. https://doi.org/10.1016/j.chemosphere.2021.132736 10.1016/j.chemosphere.2021.132736 34728224

[15] Tesfaye G. Tessema M. Negash N. Electrochemical determination of vitamin B6 in pharmaceutical and energy drink samples. Journal of Electrochemical Science and Engineering 13(13) (2023) 297-319. https://doi.org/10.5599/jese.1674 10.5599/jese.1674

[16] Gupta Y. Ghrera A.S. Development of conducting paper-based electrochemical biosensor for procalcitonin detection. ADMET and DMPK 11(11) (2023) 263-275. https://doi.org/10.5599/admet.1575 10.5599/admet.1575 37325120 PMC10262228

[17] Raril C. Manjunatha J.G. Ravishankar D.K. Fattepur S. Siddaraju G. Nanjundaswamy L. Validated electrochemical method for simultaneous resolution of tyrosine, uric acid, and ascorbic acid at polymer modified nano-composite paste electrode. Surface Engineering and Applied Electrochemistry 56 (2020) 415-426. https://doi.org/10.3103/S1068375520040134 10.3103/S1068375520040134.

[18] Borji S. Beitollahi H. Nejad F.G. Evaluating the Electrochemical Detection of Methyldopa in the Presence of Hydrochlorothiazide using a modified Carbon Paste Electrode and Voltammetric Analysis. Topics in Catalysis 67(67) (2024) 773-784. https://doi.org/10.1007/s11244-023-01855-y 10.1007/s11244-023-01855-y

[19] Mohanraj J. Durgalakshmi D. Rakkesh R.A. Balakumar S. Rajendran S. Karimi-Maleh H. Facile synthesis of paper based graphene electrodes for point of care devices: A double stranded DNA (dsDNA) biosensor. Journal of Colloid and Interface Science 566 (2020) 463-472. https://doi.org/10.1016/j.jcis.2020.01.089 10.1016/j.jcis.2020.01.089 32032811

[20] Manjunatha J.G. Raril C. Hareesha N. Charithra M.M. Pushpanjali P.A. Tigari G. Ravishankar D.K. Mallappaji S.C. Gowda J. Electrochemical fabrication of poly (niacin) modified graphite paste electrode and its application for the detection of riboflavin. The Open Chemical Engineering Journal 31 (2020) 1. https://doi.org/10.2174/1874123102014010090 10.2174/1874123102014010090

[21] Tajik S. Shams P. Beitollahi H. Garkani Nejad F. Electrochemical Nanosensor for the Simultaneous Determination of Anti-cancer Drugs Epirubicin and Topotecan Using UiO-66-NH2/GO Nanocomposite Modified Electrode. Biosensors 14(14) (2024) 229. https://doi.org/10.3390/bios14050229 10.3390/bios14050229 38785703 PMC11117627

[22] Prasanna S.B. Bahajjaj A.A.A. Lee Y.H. Lin Y.C. Dhawan U. Sakthivel R. Chung R.J. Highly responsive and sensitive non-enzymatic electrochemical sensor for the detection of β-NADH in food, environmental and biological samples using AuNP on polydopamine/titanium carbide composite. Food Chemistry 426 (2023) 136609. https://doi.org/10.1016/j.foodchem.2023.136609 10.1016/j.foodchem.2023.136609 37331138

[23] Tao B. Yang W. Miao F. Zang Y. Chu P.K. A sensitive enzyme-free electrochemical sensor composed of Co3O4/CuO@ MWCNTs nanocomposites for detection of L-lactic acid in sweat solutions. Materials Science and Engineering: B 288 (2023) 116163. https://doi.org/10.1016/j.mseb.2022.116163 10.1016/j.mseb.2022.116163

[24] Pushpanjali P.A. Manjunatha J.G. Amrutha B.M. Hareesha N. Development of carbon nanotube-based polymer-modified electrochemical sensor for the voltammetric study of Curcumin. Materials Research Innovations 25 (2021) 412-420. https://doi.org/10.1080/14328917.2020.1842589 10.1080/14328917.2020.1842589

[25] Basande A. Beitollahi H. Electrocatalytic response of nitrogen-doped hollow carbon spheres modified glassy carbon electrode for sulphite detection in water. Journal of Electrochemical Science and Engineering 13(13) (2023) 937-948. https://doi.org/10.5599/jese.1966 10.5599/jese.1966

[26] Charithra M.M. Manjunatha J.G. Electrochemical sensing of paracetamol using electropolymerised and sodium lauryl sulfate modified carbon nanotube paste electrode. ChemistrySelect 5(5) (2020) 9323-9329. https://doi.org/10.1002/slct.202002626 10.1002/slct.202002626

[27] Cheraghi S. Taher M.A. Karimi-Maleh H. Karimi F. Shabani-Nooshabadi M. Alizadeh M. Al-Othman A. Erk N. Raman P.K.Y. Karaman C. Novel enzymatic graphene oxide based biosensor for the detection of glutathione in biological body fluids. Chemosphere 287 (2022) 132187. https://doi.org/10.1016/j.chemosphere.2021.132187 10.1016/j.chemosphere.2021.132187 34509007

[28] Tigari G. Manjunatha J.G. Poly (glutamine) film-coated carbon nanotube paste electrode for the determination of curcumin with vanillin: an electroanalytical approach. Monatshefte für Chemie-Chemical Monthly 151 (2020) 1681-1688. https://doi.org/10.1007/s00706-020-02700-8 10.1007/s00706-020-02700-8

[29] Charithra M.M. Manjunatha J.G. Electrochemical sensing of adrenaline using surface modified carbon nanotube paste electrode. Materials Chemistry and Physics 262 (2021) 124293. https://doi.org/10.1016/j.matchemphys.2021.124293 10.1016/j.matchemphys.2021.124293

[30] Manjunatha J.G. Highly sensitive polymer based sensor for determination of the drug mitoxantrone. J. Surface Sci. Technol. 34 (2018) 74-80. https://doi.org/10.18311/jsst/2018/15838 10.18311/jsst/2018/15838

[31] Sarbandian Z. Beitollahi H. An electrochemical sensor based on a modified glassy carbon electrode for detection of epinephrine in the presence of theophylline. ADMET and DMPK 12(12) (2024) 391-402. https://doi.org/10.5599/admet.2082 10.5599/admet.2082 38720927 PMC11075160

[32] Beitollahi H. Tajik S. Mohammadi S.Z. Baghayeri M. Voltammetric determination of hydroxylamine in water samples using a 1-benzyl-4-ferrocenyl-1H-[1, 2, 3]-triazole/carbon nanotube-modified glassy carbon electrode. Ionics 20 (2014) 571-579. https://doi.org/10.1007/s11581-013-1004-0 10.1007/s11581-013-1004-0

[33] Zhong W. Zou J. Yu Q. Gao Y. Qu F. Liu S. Zhou H. Lu L. Ultrasensitive indirect electrochemical sensing of thiabendazole in fruit and water by the anodic stripping voltammetry of Cu^2+^ with hierarchical Ti_3_C_2_T_x_-TiO_2_ for signal amplification. Food Chemistry 402 (2023) 134379. https://doi.org/10.1016/j.foodchem.2022.134379 10.1016/j.foodchem.2022.134379 36179525

[34] Zhang Y. Li N. Xu Y. Yang M. Luo X. Hou C. Huo D. An ultra-sensitive electrochemical aptasensor based on Co-MOF/ZIF-8 nano-thin-film by the in-situ electrochemical synthesis for simultaneous detection of multiple biomarkers of breast cancer. Microchemical Journal 187 (2023) 108316. https://doi.org/10.1016/j.microc.2022.108316 10.1016/j.microc.2022.108316

[35] Guo M. Zhu G. Mishchenko Y. Butenko A. Kovalenko V. Rozhkova T. Zhao H. Highly sensitive electrochemical detection of gallic acid in tea samples by using single-walled carbon nanotubes@ silica dioxide nanoparticles decorated electrode. International Journal of Electrochemical Science 18(18) (2023) 100291. https://doi.org/10.1016/j.ijoes.2023.100291 10.1016/j.ijoes.2023.100291

[36] Maseed H. Reddy Yenugu V.M. Devarakonda S.S. Petnikota S. Gajulapalli M. Srikanth V.V. Peroxidase-like Fe3O4 nanoparticle/few-layered graphene composite for electrochemical detection of dopamine, ascorbic acid, and uric acid. ACS Applied Nano Materials 6(6) (2023) 18531-18538. https://doi.org/10.1021/acsanm.3c04018 10.1021/acsanm.3c04018

[37] Manjunatha J.G. A promising enhanced polymer modified voltammetric sensor for the quantification of catechol and phloroglucinol. Analytical and Bioanalytical Electrochemistry 12(12) (2020) 893-903.

[38] Zhang Z. Karimi-Maleh H. In situ synthesis of label-free electrochemical aptasensor-based sandwich-like AuNPs/PPy/Ti_3_C_2_Tx for ultrasensitive detection of lead ions as hazardous pollutants in environmental fluids. Chemosphere 324 (2023) 138302. https://doi.org/10.1016/j.chemosphere.2023.138302 10.1016/j.chemosphere.2023.138302 36871797

[39] Saranya J. Sreeja B.S. Senthil Kumar P. Microwave assisted cisplatin-loaded CeO_2_/GO/c-MWCNT hybrid as drug delivery system in cervical cancer therapy. Applied Nanoscience 13(13) (2023) 4219-4233. https://doi.org/10.1007/s13204-023-02856-9 10.1007/s13204-023-02856-9

[40] Bijad M. Karimi-Maleh H. Farsi M. Shahidi S.A. An electrochemical-amplified-platform based on the nanostructure voltammetric sensor for the determination of carmoisine in the presence of tartrazine in dried fruit and soft drink samples. Journal of Food Measurement and Characterization 12(12) (2018) 634-640. https://doi.org/10.1007/s11694-017-9676-1 10.1007/s11694-017-9676-1

[41] Zhang Y. Zhang B. Li J. Liu J. Huo X. Kang F. SnSe nano-particles as advanced positive electrode materials for rechargeable aluminum-ion batteries. Chemical Engineering Journal 403 (2021) 126377. https://doi.org/10.1016/j.cej.2020.126377 10.1016/j.cej.2020.126377

[42] Ozturk D. Fe_3_O_4_/Mn_3_O_4_/ZnO-rGO hybrid quaternary nano-catalyst for effective treatment of tannery wastewater with the heterogeneous electro-Fenton process: Process optimization. Science of the Total Environment 828 (2022) 154473. https://doi.org/10.1016/j.scitotenv.2022.154473 10.1016/j.scitotenv.2022.154473 35278567

[43] Sheikh A. Abourehab M.A. Tulbah A.S. Kesharwani P. Aptamer-grafted, cell membrane-coated dendrimer loaded with doxorubicin as a targeted nanosystem against epithelial cellular adhesion molecule (EpCAM) for triple negative breast cancer therapy. Journal of Drug Delivery Science and Technology 86 (2023) 104745. https://doi.org/10.1016/j.jddst.2023.104745 10.1016/j.jddst.2023.104745

[44] Manjunatha JG. Fabrication of efficient and selective modified graphene paste sensor for the determination of catechol and hydroquinone. Surfaces 3(3) (2020) 473-83. https://doi.org/10.3390/surfaces3030034 10.3390/surfaces3030034

[45] Venkateswarlu S. Mahajan H. Panda A. Lee J. Govindaraju S. Yun K. Yoon M. Fe_3_O_4_ nano assembly embedded in 2D-crumpled porous carbon sheets for high energy density supercapacitor. Chemical Engineering Journal 420 (2021) 127584. https://doi.org/10.1016/j.cej.2020.127584 10.1016/j.cej.2020.127584

[46] Luu T.V.H. Tho N.T.M. Thuy T.T.T. Thong L.N. Dung N.T. Dang P.H., Synthetization pill-like C-doped ZnO nano-photocatalyst for removing ofloxacin and methylene blue under visible light. Journal of Sol-Gel Science and Technology 110(110) (2024) 204-220. https://doi.org/10.1007/s10971-024-06348-2 10.1007/s10971-024-06348-2

[47] Song H. Peng Y. Wang C. Shu L. Zhu C. Wang Y. He H. Yang W. Structure regulation of MOF nanosheet membrane for accurate H_2_/CO_2_ separation. Angewandte Chemie, 135(135) (2023) 202218472. https://doi.org/10.1002/ange.202218472 10.1002/ange.202218472 36854948

[48] Peng F. Zhang Z. Sun M. Shao Y. Feng Y. evaluating performance of nano-Fe_3_O_4_ modified granular activated carbon assisted wastewater treatment in anaerobic fluidized membrane bioreactor. Bioresource Technology 374 (2023) 128737. https://doi.org/10.1016/j.biortech.2023.128737 10.1016/j.biortech.2023.128737 36781146

[49] Pompeu L.D. Muraro P.C.L. Chuy G. Vizzotto B.S. Pavoski G. Espinosa D.C.R. da Silva Fernandes L. da Silva W.L. Adsorption for rhodamine b dye and biological activity of nano-porous chitosan from shrimp shells. Environmental Science and Pollution Research 29(29) (2022) 49858-49869. https://doi.org/10.1007/s11356-022-19259-y 10.1007/s11356-022-19259-y 35220543

[50] Beitollahi H. Shahsavari M. Sheikhshoaie I. Tajik S. Jahani P.M. Mohammadi S.Z. Afshar A.A. Amplified electrochemical sensor employing screen-printed electrode modified with Ni-ZIF-67 nanocomposite for high sensitive analysis of Sudan I in present bisphenol A. Food and Chemical Toxicology 161 (2022) 112824. https://doi.org/10.1016/j.fct.2022.112824 10.1016/j.fct.2022.112824 35101579

[51] Karaman C. Karaman O. Show P.L. Orooji Y. Karimi-Maleh H. Utilization of a double-cross-linked amino-functionalized three-dimensional graphene networks as a monolithic adsorbent for methyl orange removal: equilibrium, kinetics, thermodynamics and artificial neural network modeling. Environmental Research 207 (2022) 112156. https://doi.org/10.1016/j.envres.2021.112156 10.1016/j.envres.2021.112156 34599897

[52] Shahsavari M. Tajik S. Sheikhshoaie I. Nejad F.G. Beitollahi H. Synthesis of Fe_3_O_4_@ copper (II) imidazolate nanoparticles: Catalytic activity of modified graphite screen printed electrode for the determination of levodopa in presence of melatonin. Microchemical Journal 170 (2021) 106637. https://doi.org/10.1016/j.microc.2021.106637 10.1016/j.microc.2021.106637

[53] Pareek S. Jain U. Bharadwaj M. Saxena K. Roy S. Chauhan N. An ultrasensitive electrochemical DNA biosensor for monitoring Human papillomavirus-16 (HPV-16) using graphene oxide/Ag/Au nano-biohybrids. Analytical Biochemistry 663 (2023) 115015. https://doi.org/10.1016/j.ab.2022.115015 10.1016/j.ab.2022.115015 36496002

[54] Zhang Z. Karimi-Maleh H. Label-free electrochemical aptasensor based on gold nanoparticles/titanium carbide MXene for lead detection with its reduction peak as index signal. Advanced Composites and Hybrid Materials 6(6) (2023) 68. https://doi.org/10.1007/s42114-023-00652-1 10.1007/s42114-023-00652-1

[55] Turan H.E. Medetalibeyoglu H. Polat I. Yola B.B. Atar N. Yola M.L. Graphene quantum dots incorporated NiAl_2_O_4_ nanocomposite based molecularly imprinted electrochemical sensor for 5-hydroxymethyl furfural detection in coffee samples. Analytical Methods 15(15) (2023) 1932-1938. https://doi.org/10.1039/D3AY00382E 10.1039/D3AY00382E 37013684

[56] Khan M.M. Shaikh H. Al Souwaileh A. Khan M.Y. Batool M. Memon S.Q. Solangi A.R. A highly selective nickel-aluminum layered double hydroxide nanostructures based electrochemical sensor for detection of pentachlorophenol. Arabian Journal of Chemistry 17(17) (2024) 105604. https://doi.org/10.1016/j.arabjc.2024.105604 10.1016/j.arabjc.2024.105604

[57] Thenrajan T. Nagappan S. Kundu S. Wilson J. Nickel iron based layered double hydroxides as effective electrochemical sensor towards epicatechin. Inorganic Chemistry Communications 153 (2023) 110861. https://doi.org/10.1016/j.inoche.2023.110861 10.1016/j.inoche.2023.110861

[58] Joseph X.B. Baby J.N. Wang S.F. George M., Emerging carbonate anion intercalated-ZnCr-layered double hydroxide/vanadium carbide nanocomposite: A electrochemical sensor for diethofencarb fungicide monitoring. Chemosphere. 335 (2023) 139099. https://doi.org/10.1016/j.chemosphere.2023.139099 10.1016/j.chemosphere.2023.139099 37270040

[59] Sohrabi H. Dezhakam E. Khataee A. Nozohouri E. Majidi M.R. Mohseni N. Trofimov E. Yoon Y. Recent trends in layered double hydroxides based electrochemical and optical (bio) sensors for screening of emerging pharmaceutical compounds. Environmental Research 211 (2022) 113068. https://doi.org/10.1016/j.envres.2022.113068 10.1016/j.envres.2022.113068 35283073

[60] Zhang L. Han Y. Sun M. Li S. Non-enzymatic electrochemical sensor based on ionic liquid [BMIM][PF6] functionalized zirconium-copper bimetallic MOF composite for the detection of nitrite in food samples. Food Chemistry 456 (2024) 140023. https://doi.org/10.1016/j.foodchem.2024.140023 10.1016/j.foodchem.2024.140023 38878537

[61] Xue C. Jamal R. Abdiryim T. Liu X. Liu F. Xu F. Cheng Q. Tang X. Fan N. An ionic liquid-modified PEDOT/Ti_3_C_2_TX based molecularly imprinted electrochemical sensor for pico-molar sensitive detection of L-Tryptophan in milk. Food Chemistry 449 (2024) 139114. https://doi.org/10.1016/j.foodchem.2024.139114 10.1016/j.foodchem.2024.139114 38581782

[62] Xhakaza N.M. Chokkareddy R. Redhi G.G. Ionic liquid based electrochemical sensor for the detection of efavirenz. Journal of Molecular Liquids 368 (2022) 120444. https://doi.org/10.1016/j.molliq.2022.120444 10.1016/j.molliq.2022.120444

[63] Zhang Z. Zheng H. Liu Y. Ma S. Feng Q. Qu J. Zhu X. Highly sensitive detection of multiple antiviral drugs using graphitized hydroxylated multi-walled carbon nanotubes/ionic liquids-based electrochemical sensors. Environmental Research 249 (2024) 118466. https://doi.org/10.1016/j.envres.2024.118466 10.1016/j.envres.2024.118466 38354882

[64] Beitollahi H. Garkani Nejad F. Dourandish Z. Aflatoonian M.R. Electrochemical detection of carmoisine in the presence of tartrazine on the surface of screen printed graphite electrode modified with nickel-cobalt layered double hydroxide ultrathin nanosheets. Chemosphere 337 (2023) 139369. https://doi.org/10.1016/j.chemosphere.2023.139369 10.1016/j.chemosphere.2023.139369 37392790

